# Extra-Adrenal Myelolipoma Containing Small Lymphocytic Lymphoma/Chronic Lymphocytic Leukemia: A Case Report and Review of the Literature

**DOI:** 10.1155/2016/7364951

**Published:** 2016-03-29

**Authors:** Komal Arora, Jagmohan Sidhu

**Affiliations:** ^1^The University of Texas MD Anderson Cancer Center, Houston, TX 77030, USA; ^2^UHS Wilson Medical Center, 33-57 Harrison Street, Johnson City, NY 13790, USA

## Abstract

Myelolipoma is a benign tumor consisting of mature fat interspersed with hematopoietic elements resembling bone marrow. The vast majority occurs within the adrenal glands, but several cases of extra-adrenal myelolipomas (EAMLs) have been reported. We report a case of a 64-year-old male who presented with complaint of lower abdominal discomfort. CT scan of abdomen and pelvis showed a 6 cm × 5 cm, well-circumscribed, predominantly fatty mass in the presacral region. Histological examination of the pelvic mass revealed a myelolipoma heavily infiltrated by small lymphoid cell aggregates with immunophenotypic features of small lymphocytic lymphoma/chronic lymphocytic leukemia (SLL/CLL). Review of the literature revealed that there is only one published report of SLL/CLL involving a myelolipoma, which was also an extra-adrenal myelolipoma, and, therefore, our case is the second case of a SLL/CLL involving a myelolipoma that is an extra-adrenal myelolipoma. Extra-adrenal myelolipomas seem to the preferred myelolipomas for involvement by SLL/CLL.

## 1. Introduction

Myelolipomas are benign tumors, which most commonly occur in adrenal gland and are composed of a mixture of hematopoietic cells and adipose tissue. They are usually nonfunctioning asymptomatic adrenal tumors and often found incidentally on radiographic studies [1]. Extra-adrenal myelolipoma can cause tumoral mass effects on adjacent organs, or it is detected incidentally during routine work-up. Hemorrhage, hematoma formation, or rupture may occur in massive tumoral involvement [2]. Surgical treatment becomes necessary when the tumor is functional or increases in size or becomes symptomatic. The purpose of this case report is to report a second case of SLL/CLL involving an extra-adrenal myelolipoma.

## 2. Case Report

A 64-year-old male presented with complaint of lower abdominal discomfort. Computerized tomographic (CT) scan revealed a retroperitoneal pelvic mass measuring 2 cm, which was radiologically considered to be a lipoma. The patient refused surgery and wanted to wait and watch. A yearly CT scan did not show any significant change in the mass for 6 years. A CT scan done in the seventh year showed increase in the size of this mass to about 6 cm × 5 cm. The patient subsequently underwent a colonoscopy which was unremarkable. He was then referred for surgery and the mass was surgically removed to rule out sarcoma. A complete blood count with differential leukocyte count was done before surgery. A positron emission tomography-computerized tomography (PET-CT) scan, bone marrow aspirate, and bone marrow core biopsy were done after the microscopic diagnosis of the resected mass. Other studies that were performed included serum protein electrophoresis, serum immunofixation electrophoresis, flow cytometric analysis of the bone marrow and peripheral blood, cytogenetic analysis of the marrow, a polymerase chain reaction (PCR) on the excised presacral mass to determine the B-cell clonality, and fluorescence in situ hybridization (FISH) to look for the common cytogenetic abnormalities found in SLL/CLL.

## 3. Pathological Findings

**Figure 1:**
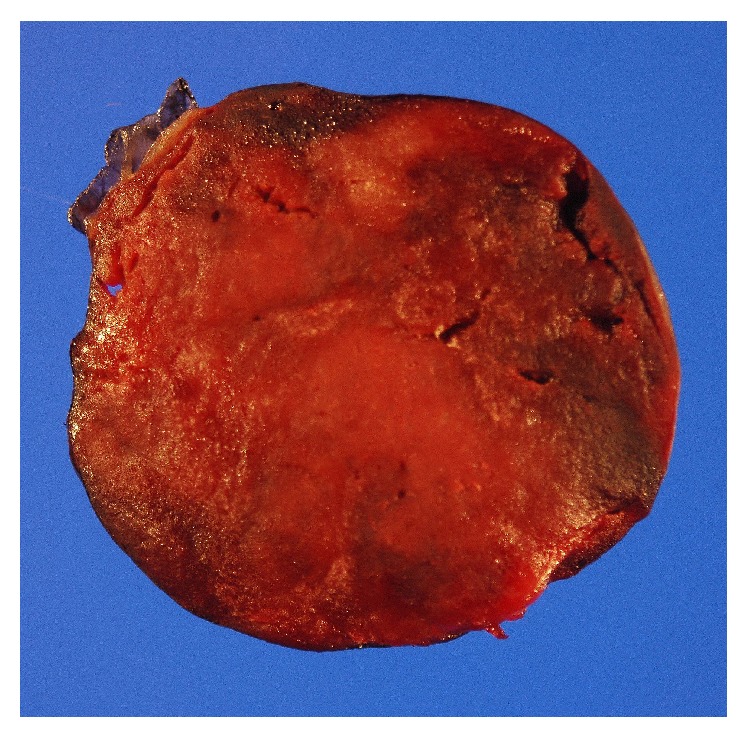
Gross photograph showing encapsulated tumor with fatty cut surface and areas of red brown discoloration.

**Figure 2:**
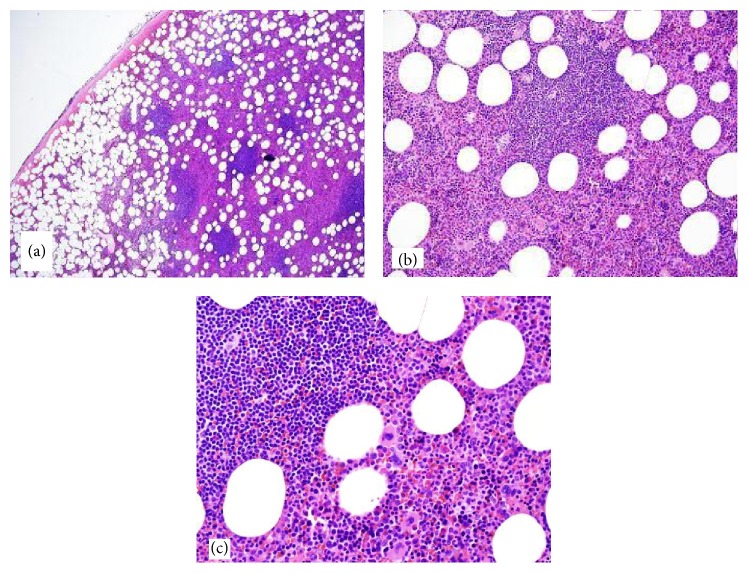
Tumor composed of mature adipose tissue intermixed with marrow elements and foci of lymphoid aggregates. (a) Scanner view, (b) low power, and (c) medium power.

**Figure 3:**
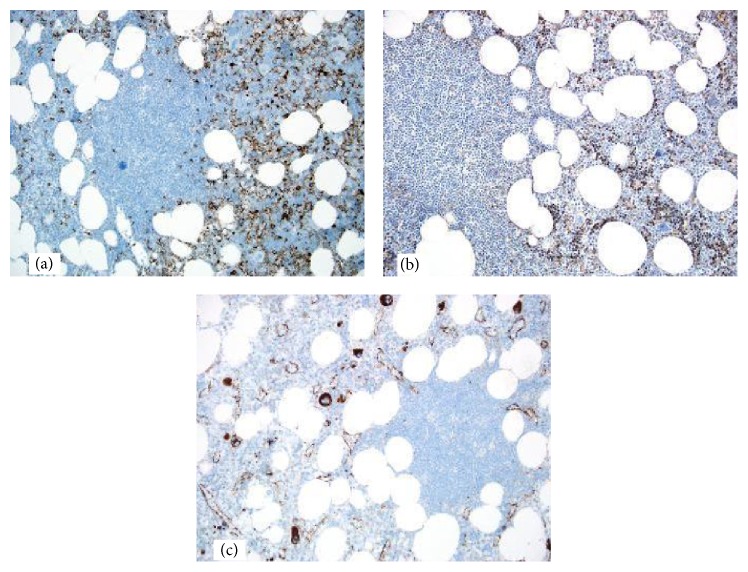
Immunohistochemical stains showing presence of hematopoietic elements comprising (a) myeloperoxidase positive myeloid component, (b) hemoglobin A-positive erythroid precursors, and (c) factor VIII-positive megakaryocytes.

**Figure 4:**
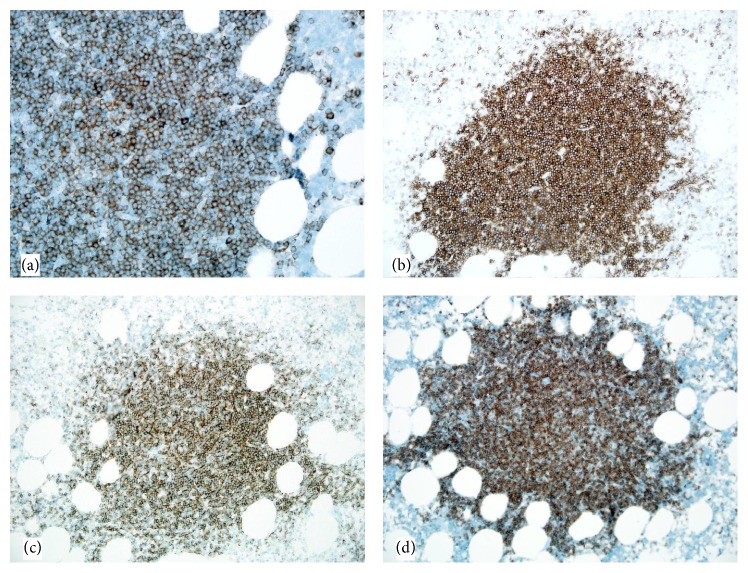
Lymphoid aggregates showing immunoreactivity for (a) CD79a, (b) CD5, (c) CD23, and (d) BCL2.

**Figure 5:**
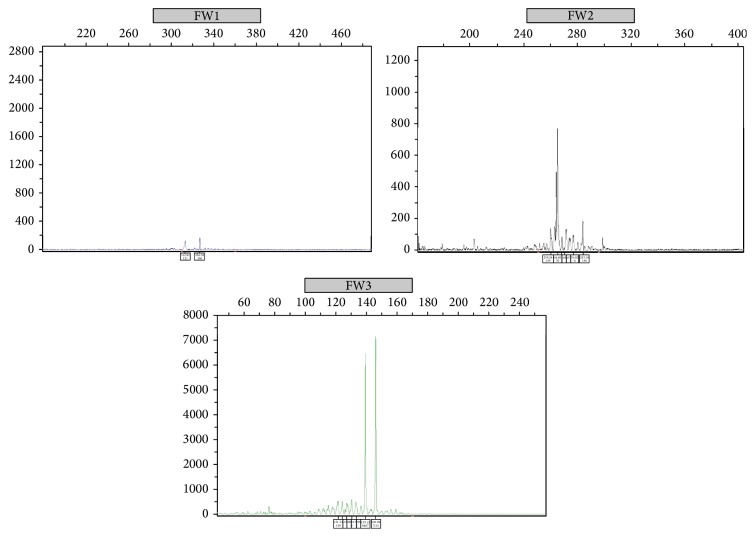
Clonal rearrangement of the immunoglobulin heavy chain (IgH) gene using BIOMED-2 multiplex PCR primers. A clonal rearrangement is detected with two of three primer sets (FR II and FR III).

Grossly the mass measured 5.7 cm × 5.2 cm × 4.2 cm. It was yellow tan to red brown, globular, and encapsulated and showed a fatty cut surface with foci of reddish-brown discoloration (Figure 1). Microscopic examination of hematoxylin and eosin stained sections demonstrated a mass composed of adipose tissue, bone marrow elements of all three cell lineages, and numerous small lymphocytic aggregates (Figure 2). A connective tissue capsule surrounded most of the mass, but the lymphoid and myeloid elements were reaching the margin of excision. Paraffin section immunohistochemical stains confirmed the presence of hematopoietic elements comprising myeloperoxidase and CD43-positive myeloid component, hemoglobin A-positive erythroid precursors, and factor VIII-positive megakaryocytes (Figure 3). CD34 immunostain revealed presence of about 2% myeloblasts in the myeloid component. Ki-67 positivity was seen in the proliferating hematopoietic elements. Some of the myeloid cells showed CD10 positivity. The lymphoid aggregates showed predominance of small B-cells which were positive for LCA, CD20 (very dim intensity), CD79a, CD5, CD23, and BCL2 (Figure 4). A few CD3 positive T-cells were also admixed with the B-cells. Ki-67 labeling index was 2% in the lymphoid component. Both myeloid and lymphoid components were negative for BCL1. In situ hybridization for kappa and lambda light chain mRNA showed very small number of polytypic plasma cells. B-cell aggregates did not show any evidence of monotypicality by in situ hybridization for light chain mRNA. A PCR study done on the mass using BIOMED-2 multiplex primers for immunoglobulin heavy chain gene rearrangement (IgH) revealed a clonal rearrangement of the IgH gene (Figure 5).

The PET scan did not reveal any evidence of residual tumor or hypermetabolic areas anywhere. Laboratory studies of peripheral blood revealed hemoglobin of 13.8 g/dL, hematocrit 40.1%, MCV 84.6 fL, platelet count 251,000/*μ*L, WBC 9,500/*μ*L with 74% neutrophils, 16% lymphocytes, 7% monocytes, 2% eosinophils, and 1% basophils. Peripheral blood film confirmed the hemogram findings. No absolute lymphocytosis was present and occasional lymphocytes showed hypercondensed chromatin. Bone marrow aspirate and touch imprints revealed normoblastic erythroid maturation, normal number and morphology of megakaryocytes, normal myeloid series, 20% lymphoid cells, and M : E ratio of 2.8 : 1. Clot section of bone marrow aspirate revealed an overall cellularity of about 70% and very few small lymphocytic aggregates. Bone marrow core biopsy did not reveal any lymphoid aggregates. Immunohistochemical stains done on clot section revealed small lymphocytic aggregates showing CD20 (very dim intensity), CD5, CD23, CD79a, and BCL2 positivity. A few CD3 and CD5 positive reactive T-cells were also present. The lymphoid cells were negative for BCL1 and CD10. A few small clusters of CD138 positive plasma cells were also present. In situ hybridization for kappa and lambda light chain mRNA showed 5% monotypic lambda plasma cells.

Flow cytometric analysis of the peripheral blood revealed 2% monotypic lambda B-cells. CD20 and surface lambda light chain expression was dim. The B-cells also expressed CD19, CD5, CD23, CD43, CD22, CD25, HLA-DR, CD79b (dim), and FMC-7 (dim). Flow cytometric analysis of the bone marrow aspirate revealed 4% monotypic lambda B-cells, which expressed CD5, CD19, CD22, CD23, CD25, and HLA-DR. B-cells also showed dim expression of CD20 and surface lambda light chain. A small number of immunophenotypically normal T-cells and NK-cells were also present. No plasma cells or increased number of myeloblasts were detected.

Serum protein electrophoresis showed a monoclonal protein band in the gamma region. Serum immunofixation electrophoresis revealed the presence of IgG lambda monoclonal paraprotein band. Serum IgG and IgA levels were normal and IgM levels were slightly decreased. The above features were consistent with monoclonal gammopathy of uncertain significance.

Cytogenetic analysis of the bone marrow revealed a normal 46, XY karyotype. FISH analysis of bone marrow was done for translocation involving* CCND1* and IgH, trisomy 12, 13q14 deletion, p53 deletion/amplification, and deletion 11q22.3. However the results did not reveal evidence of any of these five cytogenetic abnormalities tested.

The combined morphologic picture, immunohistochemical features, blood and bone marrow findings, flow cytometric analysis, and molecular diagnostic findings were consistent with a myelolipoma infiltrated by small lymphocytic lymphoma/chronic lymphocytic leukemia (WHO Classification, SLL/CLL). As the bone marrow-like component in a myelolipoma is an extramedullary tissue and, in our case, it shows innumerable aggregates, not just a few aggregates, of small lymphoid cells with immunophenotypic features of SLL/CLL, we diagnosed that it has SLL/CLL and not just a monoclonal B lymphocytosis (MBL).

## 4. Comment

Most of the myelolipomas present in the adrenal gland and are well-circumscribed lesions that contain mature adipose tissue intermixed with mature hematopoietic elements. Myelolipomas were first described in 1905 by Gienke [3]. Only about 50 cases of extra-adrenal myelolipomas have been reported so far in the literature [4]. The pathogenesis of myelolipoma remains obscure. One theory by Collins suggests that a myelolipoma represents a site of extramedullary haematopoiesis [5]. Other theories proposed include development from embryonic mesenchymal rests in the adrenal glands, development from hematogenously seeded bone marrow emboli, and metaplasia of reticuloendothelial cells [6]. The most widely accepted theory, as cited by Meaglia and Schmidt in a 1992 study of the natural history of adrenal myelolipoma, is the existence of metaplasia of the reticuloendothelial cells of blood capillaries in the adrenal gland in response to stimuli such as necrosis, infection, stress, or long-term ACTH stimulation [7]. Clonality in myelolipomas has been demonstrated [8, 9]. The exact pathogenesis of extra-adrenal myelolipoma is not well known. It has been postulated that they may be the result of misplaced myeloid cells during embryogenesis followed by hyperplasia [10].

The appearance of myelolipoma on imaging is based on the fat content of the lesion. At ultrasound, it has heterogeneous echogenicity due to its typically nonuniform architecture. CT scan demonstrates large amounts of fat with areas of interspersed higher attenuation tissue. At magnetic resonance imaging, predominantly fatty areas appear as increased signal intensity on T1-weighted images and moderate hyperintensity due to the presence of marrow-like elements in the corresponding regions on T2-weighted images. The imaging appearance of myelolipoma is altered by the presence of hemorrhage. Extra-adrenal myelolipoma usually has imaging features identical to those of its adrenal counterpart, but sometimes its imaging appearance can be similar to that of liposarcoma, and a needle biopsy can be used to distinguish between these entities [2].

Extra-adrenal myelolipoma is distinct from true bone marrow in that no reticular sinusoids or bone spicules are present. Another tumor containing hematopoietic cells, occurring rarely in the retroperitoneum, is termed a localized extramedullary myeloid tumor. Extramedullary myeloid tumors are distinguished from myelolipomas by the presence of diffuse extramedullary hematopoiesis, splenomegaly, or other organomegaly, chronic anemia, and marked hyperplasia of the bone marrow [11]. Histologically, extramedullary myeloid tumor demonstrates a prominence of hematopoietic elements rather than fat, with erythroid hyperplasia and an absence of lymphoid aggregates. Previously one case each of small lymphocytic lymphoma in a pelvic myelolipoma and Hodgkin lymphoma within an adrenal myelolipoma has been reported [12, 13]. This is the second case report of a myelolipoma infiltrated by small lymphocytic lymphoma/chronic lymphocytic leukemia. The presence of increased lymphoid aggregates has been previously reported by some authors but the exact nature of these lymphoid cells was never well established [11, 14]. In another study flow cytometric analysis was performed on a presacral extra-adrenal myelolipoma with increased lymphoid aggregates. The results of the study demonstrated unremarkable T cells, polyclonal B cells, and a small fraction of NK cells. The authors concluded that these lymphoid aggregates are a benign finding, without phenotypic aberrations or B-cell monoclonality [15]. Whether the coexistence of small lymphocytic lymphoma in myelolipoma represents malignant transformation of the lymphoid component of myelolipoma or the myelolipoma is being colonized by a relatively common systemic hematopoietic malignancy (SLL/CLL); producing a collision tumor is unknown. It is possible that the microenvironment in the bone-marrow-like tissue in a myelolipoma is similar to the bone marrow microenvironment and the SLL/CLL secondarily involves the myelolipoma because of its affinity to bone marrow-like microenvironment. Lymphoma cells are derived from normal lymphoid cells and, therefore, their metastatic behavior is probably based on mechanisms of migration of these normal lymphoid cells, involving certain homing molecules and receptors. The role of lymphoid cell homing molecules and receptors in the microenvironment of myelolipoma needs to be explored further.

We have called it SLL/CLL involving a myelolipoma instead of just a MBL [16] involving a myelolipoma because of a very heavy infiltration of myelolipoma by SLL/CLL aggregates. We have recommended continuous follow-up of this patient with complete blood count and differential leukocyte count in order to watch for development of absolute lymphocytosis, flow cytometric analysis of the peripheral blood if lymphocytosis develops in the future, physical examination for palpable lymphadenopathy/organomegaly, PET/CT scan for development of internal lymphadenopathy and organomegaly, and consideration of bone marrow examination if the patient develops anemia, lymphocytosis, or thrombocytopenia during the follow-up. So far, he has not developed absolute lymphocytosis, lymphadenopathy, organomegaly, anemia, or thrombocytopenia.
